# Impact of Sex on the Nonmotor Symptoms and the Health-Related Quality of Life in Parkinson's Disease

**DOI:** 10.1155/2016/7951840

**Published:** 2016-05-16

**Authors:** Márton Kovács, Attila Makkos, Zsuzsanna Aschermann, József Janszky, Sámuel Komoly, Rita Weintraut, Kázmér Karádi, Norbert Kovács

**Affiliations:** ^1^Department of Neurology, University of Pécs, Rét Street 2, Pécs, Hungary; ^2^MTA-PTE Clinical Neuroscience MR Research Group, Rét Street 2, Pécs, Hungary; ^3^Institute of Behavioral Sciences, University of Pécs, Szigeti Street 12, Pécs, Hungary

## Abstract

*Background*. Female Parkinson's disease (PD) patients seem to experience not only more severe motor complications and postural instability but also more pronounced depression, anxiety, pain, and sleep disturbances.* Objective*. The aim of the present study was to evaluate the role of sex as a possible independent predictor of HRQoL in PD.* Methods*. In this cross-sectional study, 621 consecutive patients treated at the University of Pécs were enrolled. Severity of PD symptoms was assessed by MDS-UPDRS, UDysRS, Non-Motor Symptoms Scale, PDSS-2, Hamilton Anxiety Scale, Montgomery-Asberg Depression Rating Scale, Lille Apathy Rating Scale, and Addenbrooke Cognitive Examination. HRQoL was assessed by PDQ-39 and EQ-5D. Multiple regression analysis was performed to estimate the PDQ-39 and EQ-5D index values based on various clinical factors.* Results*. Although females received significantly lower dosage of levodopa, they had significantly more disabling dyskinesia and worse postural instability. Anxiety, pain, sleep disturbances, and orthostatic symptoms were more frequent among females while sexual dysfunction, apathy, and daytime sleepiness were more severe among males. Women had worse HRQoL than men (EQ-5D index value: 0.620 ± 0.240 versus 0.663 ± 0.229, *p* = 0.025, and PDQ-39 SI: 27.1 ± 17.0 versus 23.5 ± 15.9, *p* = 0.010). Based on multiple regression analysis, sex was an independent predictor for HRQoL in PD.* Conclusions*. Based on our results, female sex is an independent predictor for having worse HRQoL in PD.

## 1. Introduction

Recently the nonmotor symptoms (NMS) of Parkinson's disease (PD) have been increasingly recognized as a major burden of the health-related quality of life (HRQoL) [1, 2]. In PD, the range of NMS can include high variety of problems such as mood disorders (depression and anxiety), sleep disorders, cognitive dysfunctions, dementia, autonomic dysfunctions, apathy, and fatigue [3, 4]. These NMS are not only common in patients with PD, but also usually underrecognized [5]. According to some studies, at least one NMS was reported by almost 100% of patients [6, 7], with the most common being autonomic dysfunction, mood disorders, and sleep problems [8, 9]. Although many NMS are apparent and common among de novo and nontreated PD patients, there are also some NMS that are usually considered to be secondary to pharmacotherapy, such as impulse control disorders (ICD) and psychosis. Moreover, NMS have also been reported by patient surveys to be sometimes more disabling than the motor symptoms of tremor and bradykinesia [10]. In advanced PD, the NMS can have a pattern of fluctuation similar to the motor symptoms [11, 12]. Moreover, some of the PD patients state that their nonmotor fluctuations cause a greater degree of disability and distress than the motor fluctuations [13]. This fact has been reinforced by recent studies demonstrating that in some cases the NMS have higher impact on the HRQoL than the motor symptoms do [3, 14].

Previous studies incongruently demonstrated that sex is an independent predictor for some NMS [15]. Female PD patients seem to experience not only more severe motor complications and falls but also more pronounced depression, anxiety, pain, and sleep disturbances [16–18]. On the other hand, males tend to have more pertinent apathetic symptoms and sexual dysfunction [19–21]. The role of sex being an independent predictor of HRQoL in PD is controversial. Several studies demonstrated that female sex was associated with poorer HRQoL. However, the role of gender was not unambiguous even in the studies: some of these investigations concluded that female sex was an independent negative predictor while others stated that female sex was only a nonindependent predictor of HRQoL in PD. In other words, these latter investigations suggested that not the female sex but the nonmotor symptoms associated with female sex had a direct impact on HRQoL. Indeed, several other studies have reported no gender difference for HRQoL in PD, at all, but have not specifically addressed potential reasons for this. A direct investigation of the relationship between sex and HRQoL is still lacking in a large representative pool of patients. In light of the aforementioned findings, the aim of this study was to extend recent studies investigating the association between HRQoL and disease stage in PD, motor subtype, and gender using a large pool of well-characterized patients.

## 2. Materials and Methods

### 2.1. Patients

In this cross-sectional study, 621 consecutive and nonselected patients treated at the University of Pécs were enrolled. All patients fulfilled the UK Brain Bank criteria for PD [22]. Each subject gave written informed consent in accordance with the ethical approval of the Regional Ethical Board (3617.316-24983/KK41/2009). Each patient was examined by a neurologist specialized in movement disorders. Besides recording demographic data (age, sex, and level of education) some disease-specific data were also noted (age at onset; disease duration; presence of motor complications, duration of fluctuation in years; type of PD being either tremor dominant, rigid-akinetic, or mixed type; and antiparkinsonian medication). Patients were evaluated in ON state while receiving their usual antiparkinsonian and other medications and subsequently levodopa equivalent dosage (LED) calculations were performed [23].

### 2.2. Assessment of Motor Symptoms

Severity of PD-related symptoms was globally assessed by the Hungarian validated version of the MDS-UPDRS [24, 25]. The recently published MDS-UPDRS is a validated scale to assess nonmotor (nM-EDL, Part I) and motor-experiences of daily living (M-EDL, Part II), motor examination (ME, Part III), and motor complications (MC, Part IV) [25]. As a part of the MDS-UPDRS, the Hoehn-Yahr Scale was also taken to detect the overall severity of PD. Based on previous reports, we analyzed separately items 3.12 “Postural instability” and 2.12 “Walking and balance” of MDS-UPDRS. Additionally, the axial items of MDS-UPDRS ME were also assessed following the method of Kotagal et al. [26] by summing items 3.1 “Speech,” 3.9 “Arising from a Chair,” 3.10 “Gait,” 3.12 “Postural Stability,” and 3.13 “Posture.”

Dyskinesia was measured by the Hungarian validated version of Unified Dyskinesia Rating Scale [27, 28]. We also asked our patients to keep a patient diary measuring ON state without dyskinesia, ON state with slight nondisturbing dyskinesia, ON state with severe dyskinesia, OFF state, and sleep periods for at least 3 days prior or immediately after the examination [29, 30].

### 2.3. Assessment of Nonmotor Symptoms

The nM-EDL part of the MDS-UPDRS has items evaluating the presence and severity of 13 NMS including depression, anxiety, apathy, dopamine-dysregulation, cognitive impairment, fatigue, pain, hallucinations, urinary problems, sexual problems, orthostatic problems, nighttime sleep problems, and daytime sleepiness. These items are also intended to serve as screening tools for the presence of these nonmotor symptoms [31]. To assess nonmotor symptoms globally, the Non-Motor Symptoms Scale (NMSS) [32] was also included. This scale is obtained by trained professionals and capable of simultaneously capturing the severity and frequency of 30 nonmotor symptoms typical for PD. These NMSS items can group nine domains including sleep, cardiovascular, cognitive, mood, hallucinatory, gastrointestinal, urinary, sexual, and miscellaneous problems.

Presence and severity of sleep disturbances were specifically measured by the Hungarian validated version of PDSS-2 [33, 34]. The threshold indicating sleep problems is 11 points for the Hungarian version of PDSS-2 [35]. In the meantime, daytime sleepiness was assessed by the Epworth Sleepiness Scale [36] with the cutoff value of 8 points [37]. Depression, anxiety, and apathy were assessed by the Hungarian validated versions of the Montgomery Depression Scale (MADRS) [38], the Hamilton Anxiety Scale (HAM-A) [39], and the Lille Apathy Scale (LARS) [40]. Cognitive performance was examined by the Hungarian validated versions of Mini-Mental Status Examination (MMSE) [41], Montreal Cognitive Assessment (MoCA) [42], Mattis Dementia Rating Scale (MDRS) [41], and Addenbrooke Cognitive Examination (ACE) [41, 43]. Presence of dementia (major neurocognitive disorder) was defined as either achieving ≤125 points on the Hungarian validated version of the Mattis Dementia Rating Scale [41, 43] and/or ≤22 points on the Montreal Cognitive Assessment [43] and/or fulfilling the criteria of dementia according to the DSM-5 [44]. Presence and severity of impulse control disorders (ICD) were assessed by the questionnaire for impulsive-compulsive disorders in Parkinson's disease (QUIP) [45]. The global functioning was evaluated by the Schwab & England Scale (SES) [38, 46].

Health-related quality of life (HRQoL) was measured by the Hungarian validated version of the disease-specific PDQ-39 Summary Index (PDQ-39 SI) [47] and the nondisease specific EuroQol instrument (EQ-5D index value) [48, 49]. Generally, higher scores on PDQ-39 SI and smaller index values on the EQ-5D represent worse HRQoL states.

The whole assessment required a total of approximately 5-6 hours to complete. To reduce the burden of patients, the clinical assessments were performed on three consecutive days. Typically on Day 1 both the ME part of MDS-UPDRS and UDysRS were taken. On Day 2, the patients underwent the neurocognitive evaluation by psychologists. All the patient reported questionnaires had to be completed by Day 3 (including the PD diary).

### 2.4. Statistical Analysis

All statistical analyses were carried out using the IBM SPSS software package (version 22.0.1, IBM Inc., Armonk, NY, USA). Because data from these scales followed the normal distribution, mean and standard deviations (SD) were calculated. For group comparisons, independent samples *t*-tests were applied. Evaluating significant differences in single items of MDS-UPDRS, both independent samples *t*- and Mann-Whitney tests were utilized because they were ordinal variables. For categorical variables (e.g., having or not having a symptom) Chi-square tests were used. Statistical significance level was set at 5%.

A multiple regression modelling using a stepwise method (criteria: probability of *F* to enter ≤0.050 and probability of *F* to remove ≥0.100) was initiated to predict the PDQ-39 SI from various clinical variables including sex, age, age at disease onset, years of education, disease duration, fluctuations in years, handedness, disease-type, ACE, MDRS, MADRS, levodopa dosage (measured in LED), DA LED, total LED, LARS, ON time without dyskinesia, ON time with slight dyskinesia, ON time with severe dyskinesia, OFF time, PICD, PDSS-2, ESS, HAS, UDysRS, M-EDL part of MDS-UPDRS, ME part of MDS-UPDRS, and MC part of MDS-UPDRS. Subsequently, another multiple regression analysis was performed to predict the EQ-5D index values from the same variables using a stepwise method.

## 3. Results

### 3.1. Demographic and PD-Related Clinical Data

The subject population consisted of 621 consecutive PD patients (361 males, age: 66.9 ± 9.2 years, disease duration: 7.6 ± 6.1 years). Two-hundred and forty-seven patients had rigid-akinetic, 194 had tremor dominant, and 180 had mixed type of PD. In the examined sample, 234 patients (37.7%) had motor fluctuations with the average duration of 6.5 ± 4.2 years. Only 127 (20.5%) patients had either fulltime or part-time job at the time of the exam. Handedness, dominant side, and Hoehn-Yahr staging are demonstrated in Table 1, whereas the medication usage and levodopa equivalent dosages are shown in Table 2. Levodopa treatment was applied in 454 (73.1%), dopamine-agonists in 320 (51.5%), and catechol-O-methyl-transferase inhibitors in 223 (35.9%) patients. In the studied population 98 (15.8%) patients underwent deep brain stimulator implantation with electrodes targeted into the subthalamic nuclei bilaterally.

### 3.2. Motor Symptoms of PD

Although age at PD onset, disease duration, education years, and severity of motor symptoms (MDS-UPDRS Motor Examination) were comparable between the males and females, men received significantly higher dosage of levodopa (551.4 ± 413.3 mg versus 423.6 ± 386.3 mg, *p* = 0.001, Table 2). Based on the M-EDL MDS-UPDRS, the overall motor symptoms were associated with similar disabilities in both sexes. Although the axial scores on MDS-UPDRS ME were comparable, females had significantly worse postural instability (item 3.12) and gait-related disabilities (item 2.12).

In the examined population, 144 males (39.9%) and 90 females (34.6%) had fluctuations (*p* = 0.181, Chi-square test). Despite of receiving less dopaminergic medication, women had significantly worse dyskinesia compared to men (UDysRS total score: 35.5 ± 18.6 versus 30.1 ± 17.4 points, resp., *p* = 0.006, Table 2). However, the analysis of the patient diaries revealed that both sexes had comparable ON and OFF time. The only statistically significant difference was the time of daytime sleep (males: 0.7 ± 1.2 hours versus females: 0.5 ± 0.8 hours, *p* = 0.005).

### 3.3. Nonmotor Symptoms of PD

In our study cohort only 6 patients (0.9%) did not report any NMS, at all. Based on the 13 screening items of nM-EDL part of MDS-UPDRS, our patients had an average of 8.08 ± 2.78 NMS symptoms. Female patients had more severe nonmotor symptoms in general. This finding is congruently supported by the nM-EDL part of MDS-UPDRS (15.1 ± 7.9 versus 13.8 ± 7.5 points, *p* = 0.034, Table 2) and NMSS scores (64.1 ± 41.1 versus 57.4 ± 41.2 points, *p* = 0.045, Table 2).

#### 3.3.1. Affective Problems

Among female PD patients the anxiety was not only significantly more frequent (85.0% versus 76.5%, *p* = 0.005), but also more severe (HAM-A score: 16.0 ± 6.9 versus 12.5 ± 6.0, *p* = 0.001). Although the prevalence of depression was comparable between both sexes (76.2% versus 73.7%, *p* = 0.386), the severity of depression was worse in women (MADRS score: 14.2 ± 7.6 versus 11.8 ± 8.0, *p* = 0.003) (Table 2). Similarly, the “Mood problems” section of NMSS demonstrated more severe affective problems in the female individuals (15.3 ± 12.3 versus 12.4 ± 14.3, *p* = 0.016) (Table 2).

#### 3.3.2. Sleep-Related Problems

Based on the Hungarian validated threshold values for PDSS-2, 72.7% of females and 63.4% of males reported sleep-related problems (Chi-square test, *p* = 0.034). Although the female PD patients had more severe nighttime sleep disturbances (measured by the total score of PDSS-2), daytime sleepiness was more common (39.3% versus 26.9%, *p* = 0.001) and more severe among males (Table 2).

#### 3.3.3. Cardiovascular and Orthostatic Problems

Based on the screening item of MDS-UPDRS (1.12 orthostatic symptoms) and the “Cardiovascular” section of NMSS, female patients had more often (71.5% versus 62.6%, Chi-square test, *p* = 0.023) and more severe orthostatic and cardiovascular problems than males (*p* = 0.004, Table 2).

#### 3.3.4. Sexual Problems

Male patients had more frequent (31.6% versus 18.1%, *p* < 0.001, Chi-square test) and more severe sexual problems than females (2.9 ± 5.8 versus 1.8 ± 5.2, *p* = 0.022, Table 2).

#### 3.3.5. Pain

Based on the screening item of MDS-UPDRS (1.9 Pain) and the item 27 of NMSS (Pain), women had more frequent (76.5% versus 67.3%, *p* = 0.014, Chi-square test) and more severe (4.8 ± 3.7 versus 2.1 ± 3.1, *p* < 0.001) pain sensations than men.

#### 3.3.6. Apathy

Although the prevalence of apathy was comparable (18.5% versus 21.9%, Chi-square test, *p* = 0.279), males had more severe apathetic symptoms measured by LARS than females (−20.4 ± 10.8 versus −22.8 ± 8.9, *p* = 0.004).

#### 3.3.7. Cognition

We could not demonstrate any differences in cognition either by NMSS or specific neurocognitive screening tests (MMSE, MoCA, ACE, and MDRS) (Table 2).

#### 3.3.8. Other NMS Problems

Both sexes had similarly frequent and severe urinary, gastrointestinal, and hallucinatory problems. Based on MDS-UPDRS nM-EDL, we could not find any differences in the prevalence and the degree of fatigue, either.

### 3.4. Impulse Control Disorders in PD

Based on the analysis of QUIP, 21.6% of male and 20.0% of female PD patients had any type and any degree of ICD problems (*p* = 0.850, Table 3). The prevalence and severity of pathological gambling, compulsive eating, and punding were similar in both sexes. However, men had more often hypersexuality (5% versus 0%, *p* < 0.001) and women had more frequent and severe compulsive buying (18.5% versus 6.4%, *p* < 0.001).

### 3.5. HRQoL

Although both male and female PD patients had similar everyday functioning measured by the SES (75.0 ± 16.2 versus 74.1 ± 19.4 points, *p* = 0.636, Table 2), female patients had worse HRQoL than males (EQ-5D index value: 0.620 ± 0.240 versus 0.663 ± 0.229, *p* = 0.026 and PDQ-39 SI: 27.1 ± 17.0 versus 23.5 ± 15.9, *p* = 0.010). Out of the PDQ-39 components, “Mobility,” “Emotional well-being,” “Social support,” and “Bodily discomfort” had higher impairment in women, whereas the section of “Communication” was worse in men.

### 3.6. Determinants of HRQoL in PD

Multiple regression analysis was performed to estimate the PDQ-39 SI based on various clinical factors. Due to collinearity, age of disease onset, MDRS, and MMSE scores were excluded from the regression analysis (tolerance < 0.001). The data met the assumption of independent errors (Durbin-Watson value = 1.911). Using a stepwise method it was found that M-EDL part of MDS-UPDRS (importance = 0,53; *β* = 0.883, *p* < 0.001), PDSS-2 total score (importance = 0.12; *β* = 0.260, *p* < 0.001), MADRS total score (importance = 0.09; *β* = 0.423, *p* = 0.002), sex (coded as 1 = males and 2 = females, importance = 0.06; *β* = −3.389, *p* = 0.010), ME part of MDS-UPDRS (importance = 0,05; *β* = −0.134, *p* = 0.012), SES (importance = 0.04; *β* = −0.124, *p* = 0.017), UDysRS total score (importance = 0.03; *β* = 0.166, *p* = 0.013), HAM-A total score (importance = 0.03; *β* = 0.258, *p* = 0.019), ACE total score (importance = 0.03; *β* = −0.118, *p* = 0.027), QUIP total score (importance = 0.02; *β* = 0.667, *p* = 0.029), and ESS total score (importance = 0.02; *β* = 0.186, *p* = 0.038) explain the highest significant amount of variance in the value of the PDQ-SI score (intercept = 22.433, *F*(9,593) = 120.400, *p* < 0.001, *R* ^2^_adjusted_ = 0.741). The other examined variables did not significantly contribute to the model.

For estimating the EQ-5D index value SES (importance = 0.47; *β* = 0.006, *p* < 0.001), M-EDL part of MDS-UPDRS (importance = 0.33; *β* = −0.010, *p* < 0.001), PDSS-2 total score (importance = 0.11; *β* = −0.004, *p* = 0.013), ME part of MDS-UPDRS (importance = 0.04; *β* = −0.002, *p* = 0.012), UDysRS total score (importance = 0.03; *β* = −0.162, *p* = 0.016), QUIP total score (importance = 0.03; *β* = −0.011, *p* = 0.018), MADRS total score (importance = 0.02; *β* = −0.002, *p* = 0.038), and sex (coded as 1 = males and 2 = females, importance = 0.04; *β* = 0.032, *p* = 0.010) contributed significantly to a model (intercept = 0.318, *F*(4,309) = 46,547, *p* < 0.001, *R* ^2^_adjusted_ = 0.608).

## 4. Discussion

Although the sexual differences in PD have been previously recognized, they are poorly understood. The aim of the present study was evaluate the impact of sex on the presence and severity on various PD-related symptoms and the HRQoL.

### 4.1. Motor Symptoms and Motor Complications

First, we were unable to detect any sex-related differences in the major demographic data (e.g., age at disease onset, disease duration, and education level) and overall motor performance. Although females received significantly lower dose of levodopa, the severity of motor symptoms (MDS-UPDRS ME) and the disability related to motor symptoms (MDS-UPDRS M-EDL) were comparable between both sexes. These findings are congruent with the results of several previous studies [17, 50, 51].

Despite of the lower overall levodopa dosage, women had worse dyskinesia measured by UDysRS. This finding is also in agreement with the literature [52–54].

Because previous studies demonstrated that female sex is associated with significantly worse postural instability and more frequent falls [55–57], therefore, we separately analyzed items 3.12 “Postural instability” and 2.12 “Walking and balance” of MDS-UPDRS. In these two items, women had significantly worse scores (justified by both *t*- and Mann-Whitney tests, Table 2). Item 2.12 emphasizes the need for assistance rather than the consequence (falls) that will depend on availability of help [25]. However, together with item 3.12 it reflects the postural instability, gait difficulties, and the tendency of falls. Because other items of MDS-UPDRS M-EDL and ME did not differ between man and women (data not shown), we can conclude that the worse postural instability and tendency of falls are independently more pronounced in females.

### 4.2. Nonmotor Symptoms

Based on our data, more than 99% of the patients had at least one NMS with an average number of 8 out of the 13 items screened by the MDS-UPDRS. The most prevalent NMS problems were fatigue, anxiety, depression, daytime sleepiness, and pain. These findings are congruent with the literature [58, 59].

Consistent with the previously published data, we demonstrated that anxiety [4, 16, 17], pain [4, 17, 18], nocturnal sleep difficulties, and orthostatic symptoms were more frequent among female PD patients while the prevalence of sexual dysfunction [4, 20, 21] and daytime sleepiness were more common among males [4, 60]. Although depression was similarly common among both sexes [9], the depressive symptoms were more severe in females [61, 62]. Contrarily, the apathetic symptoms were more pronounced in males despite of their similar occurrence in both sexes. In our cohort, we could not find any sex-related differences in the frequency and severity of fatigue. In the literature there are inconsistent data available, some supporting [4, 52, 63] and others disagreeing [64] with a possible link between sex and fatigue.

### 4.3. Impulse Control Disorders

In agreement with some studies [65, 66] and in opposition to other papers [67, 68], we did not find significant differences in the prevalence of global ICD symptoms between both sexes. Though total ICD frequency was similar for men and women, there were notable sex differences in the frequency of specific ICDs, with hypersexuality more common in males and compulsive buying and binge eating were more prevalent in women. These sex-related differences were previously reported [69].

### 4.4. Determinants of HRQoL in PD

The role of sex on HRQoL in PD is highly controversial. Indeed, some publications did not demonstrate any differences in HRQoL between males and females [3, 15, 57, 70–72]. These studies were performed in distinct areas (e.g., Australia [15], Eastern-Europe [3, 57, 73], Northern-Europe [74, 75], Western-Europe [72, 76], and America [77]) and utilized heterogeneous HRQoL questionnaires (e.g., PDQ-39 [3, 15], EQ-5D [73], MOS Short Form 36, SF-36 [77], and Nottingham Health Profile [74]).

In another group of reports, the observed differences between females and males with respect to HRQoL were not solely contributed to the sex because regression analyses did not reveal the independent role of sex. Klepac et al. examined 111 consecutive Croatian PD patients, but they failed to prove the role of sex (multiple regression coefficient: 37.961, *p* = 0.051) in determining HRQoL. Marras et al. demonstrated that the presence and severity of dyskinesia, but not the sex alone, predicted the HRQoL (EQ-5D) in the case of 182 PD patients enrolled into a randomized trial (CALM-PD) and completed the 4-year follow-up examination [78].

A third group of investigations did not include the “sex” as a possible predictor in their statistical analyses [1, 79–81]. Morimoto et al. examined 1200 Japanese PD patients by SF-36 questionnaire; however, they did not enter the sex as a covariate into their regression models [80]. Similarly, Qin et al. examined the HRQoL in 391 Chinese patients with early Parkinson's disease by SF-36 without entering the sex as a possible independent factor [81]. Although Santos-García and de la Fuente-Fernández aimed to investigate the role of NMS on the health-related and the perceived quality of life on the cohort of 150 nonselected PD patients, they did not analyze the independent role of sex either [1].

A fourth group of papers demonstrated that females had worse HRQoL than males and sex was an independent predictor for HRQoL [82–88]. These studies included diverse ethnical and cultural populations (e.g., Asia [82, 83, 85], Africa [88], Eastern-Europe [87], and Western-Europe [84, 86]).

We might assume heterogeneous factors behind these diverse outcomes of the aforementioned studies. One of the most important issues might be the differences in the applied outcome measures. While the majority of the examinations applied disease-specific instruments (PDQ-39, PDQ-8 or PDQL), others utilized only generic tests (SF-36, EQ-5D, or Nottingham Health Profile). Because the construct, precision, and response of these scales are highly different, the uniform and comparable interpretation of their outcomes is difficult. Another problem might be the differences in sociocultural profiles of the enrolled individuals. Because the perceived HRQoL highly depends on cultural and social attitudes of patients, some PD-related symptoms are likely to be differently interpreted by Western-European, Eastern-European, African, Asian, American, and Oceanian individuals. The disability, the reduced working capability and self-dependence associated with PD are differently understood and accepted by many cultures and societies. For example, while the HRQoL of patients with PD in Western countries is predominately affected by clinical parameters, social factors play much more pronounced role in the Eastern countries [73].

Another key factor might be the pronounced differences in the size of enrolled patient populations. While the majority of studies having negative conclusion on the independent role of sex had the typical sample size of 100–300 patients, all the larger studies (>400 patients) had positive outcome. Of note, in some negative European studies, the *p* values were indeed close to the level of statistical significance (e.g., Kadastik-Eerme et al. [3], *p* = 0.06, and Klepac et al. [89], *p* = 0.051), which might suggest the possibility of statistical underpower in the background (Type II error).

Our study clearly demonstrates on a large pool of patients that sex is an independent predictor of HRQoL in PD. This finding was assured by the utilization of both disease-specific and general HRQoL instruments. Based on the multivariate stepwise regression analysis we could show that female sex is independently associated with poorer HRQoL despite the sex-dependent profile of NMS. One of the assumptions behind this phenomenon might be the diverse effects of estrogen. Estrogen has numerous effects on dopamine neurotransmission inhibiting dopamine uptake and altering dopamine synthesis and release [90]. These effects are extremely complex and dependent on many factors, including the nature of the estrogen exposure (e.g., exposure to normal fluctuations in estrogen levels throughout the menstrual cycle, exposure to the estrogen-deficient state of menopause, or exposure through various estrogen treatment regimens). Basic research in experimental animals indicates the neuroprotective roles of estrogen over various forms of injury [91] and one of its consequences might be the greater incidence of PD in men than in women. However, sex-related differences are also identified in response to treatment of PD; for example, women have greater levodopa bioavailability [92]. This might contribute to our observation that females have comparable overall severity of motor symptoms and more pronounced dyskinesia despite of lower levodopa equivalent dosages [93].

### 4.5. Limitations of the Study

Although we endeavored to strategize this study with precision, the authors are aware of some potential limitations. One limitation may be that our study had a monocenter design instead of a more favorable multicenter one. In the role of a simultaneously primary and a tertiary center, the University of Pécs has both noncomplicated PD patients from the surrounding areas and the advanced PD patients from the nationwide primary and secondary centers. Therefore, the pool of patients are likely different from those of typical primary or secondary centers. However, in the role of a tertiary center, consider our advantage: relatively a large portion of severe (HYS 4&5) patients were also included in our study. This may have contributed to a relatively high percentage of the population featuring advanced PD and or minor/major neurocognitive disorders.

Additionally, we applied more recent and slightly different test batteries than the majority of older studies utilized. Instead of the UPDRS and PDSS, we have applied the more advantageous MDS-UPDRS and PDSS-2. Therefore, the comparison of our results with those of older studies utilizing the preceding test batteries is not straightforward and needs careful interpretation.

## 5. Conclusions

Based on our results, female sex is an independent predictor for having worse health-related quality of life in Parkinson's disease. Because the main intention of PD-care is to improve not only the symptoms of PD but also the quality of life of patients, the recognition of female sex as a negative predictor of HRQoL is of the utmost importance. In clinical setup, therefore, additional attention should be paid to female patients to achieve the best obtainable health status and HRQoL. Besides, further studies are needed to reveal the pathophysiological differences between female and male patients.

## Figures and Tables

**Table 1:** Basic characteristics of the study population (*n* = 621).

		Mean	SD	Count	Percentage
Age at PD onset (years)		59.4	11.6		

Age at PD onset (years)	<36 years			14	2.3%
	36–45 years			63	10.1%
	46–55 years			144	23.2%
	56–65 years			205	33.0%
	66–75 years			144	23.2%
	>75 years			51	8.2%

Disease duration (years)		7.6	6.1		

Disease duration (years)	0–5 years			241	38.8%
	6–10 years			186	30.0%
	11–15 years			124	20.0%
	>15 years			70	11.3%

Gender	Male			361	58.1%
	Female			260	41.9%

Handedness	Right handed			589	94.8%
	Left handed			32	5.2%

Dominant side	Right dominance			356	57.3%
	Left dominance			265	42.7%

Education (years)		12.3	3.3		

PD type	Rigid-akinetic			247	39.8%
	Tremor dominant			194	31.2%
	Mixed type			180	29.0%

Hoehn-Yahr stage	1			29	4.7%
	2			315	50.7%
	3			164	26.4%
	4			94	15.1%
	5			19	3.1%

Fluctuation (years)		6.5	4.2		

Presence of fluctuations	No			387	62.3%
	Yes			234	37.7%

Working status	Full time job			54	8.7%
	Part-time job			73	11.7%
	No job due to illness			236	37.9%
	No job unrelated to illness			258	41.7%

Housework status	Does household work independently			347	55.9%
	With others' help does household work			146	23.5%
	Fully self-dependent but does not perform housework			67	10.8%
	Partly self-dependent			43	6.9%
	Requires full support			18	2.9%

SD: standard deviation.

**Table 2:** Impact of gender on various demographic factors, medication usage, and motor- and nonmotor symptoms of Parkinson's disease.

		Gender				*p* value
		Male (*n* = 361)		Female (*m* = 260)		
		Mean or count	SD or percentage	Mean or count	SD or percentage	
Demographic	Age at PD onset (years)	59.0	11.9	60.0	11.0	0.265
	Disease duration (years)	7.7	6.1	7.5	6.2	0.736
	Education (years)	12.8	3.1	11.5	3.4	0.112

Medication	*Levodopa dosage (LED in mg)*	*551.4*	*413.3*	*423.6*	*386.3*	*<0.001*
	Dopamine agonist dosage (LED in mg)	174.4	230.8	160.6	224.4	0.455
	*Anti-Parkinson's medication (LED in mg)*	*725.8*	*594.8*	*584.7*	*424.5*	*0.001*

MDS-UPDRS	*MDS-UPDRS nM-EDL*	*13.8*	*7.5*	*15.1*	*7.9*	*0.034*
	MDS-UPDRS M-EDL	15.4	9.2	14.9	9.2	0.536
	*MDS-UPDRS 2.12 Walking*	*1.4*	*1.1*	*1.6*	*1.2*	*0.009* ^*∗*^
	MDS-UPDRS ME	37.9	17.4	37.5	17.8	0.801
	MDS-UPDRS ME axial score	6.0	3.8	6.3	4.1	0.131
	*MDS-UPDRS 3.12 Postural instability*	*1.1*	*1.3*	*1.5*	*1.4*	*0.001* ^*∗*^
	MDS-UPDRS MC	4.7	3.8	4.8	4.2	0.752
	MDS-UPDRS Total score	71.9	31.2	72.2	33.3	0.901

UDysRS	*UDysRS Part1 ON Dyskinesia*	*12.6*	*9.3*	*15.5*	*9.3*	*0.004*
	UDysRS Part2 OFF Dyskinesia	6.5	4.6	6.8	4.5	0.605
	*UDysRS Part3 Impairment*	*6.4*	*5.2*	*7.9*	*5.6*	*0.008*
	*UDysRS Part4 Disability*	*4.3*	*3.3*	*5.0*	*3.3*	*0.033*
	*UDysRS Historic subscore*	*19.2*	*11.0*	*22.4*	*11.7*	*0.010*
	*UDysRS Objective subscore*	*10.7*	*8.2*	*13.0*	*8.6*	*0.011*
	*UDysRS Total score*	*30.1*	*17.4*	*35.5*	*18.6*	*0.006*

Patient diary	ON without dyskinesia (hours)	8.9	6.2	8.9	6.3	0.897
	ON with slight Dyskinesia (hours)	2.0	3.5	1.8	2.6	0.130
	ON with severe dyskinesia (hours)	0.3	1.3	0.3	1.3	0.911
	OFF time (hours)	4.2	5.7	5.0	6.0	0.212
	Awake time (hours)	15.4	2.2	15.5	2.1	0.931
	*Daytime sleep time (hours)*	*0.7*	*1.2*	*0.5*	*0.8*	*0.005*
	Nighttime sleeping time (hours)	7.8	1.8	8.1	1.9	0.171

Sleep	*Presence of sleep problems*	*229*	*63.4%*	*189*	*72.7%*	*0.034*
	*PDSS-2 Total score*	*16.3*	*11.2*	*18.3*	*11.1*	*0.027*
	*Presence of daytime sleepiness*	*142*	*39.3%*	*70*	*26.9%*	*0.001*
	*Epworth Sleepiness Scale*	*7.8*	*5.0*	*6.4*	*4.4*	*0.000*

Affective	*Presence of affective problems*	*276*	*76.5%*	*221*	*85.0%*	*0.036*
	*MADRS Total score*	*11.8*	*8.0*	*14.2*	*7.6*	*0.003*
	*HAM-A Total score*	*12.5*	*6.0*	*16.0*	*6.9*	*0.001*

Neurocognitive	ACE Total score	82.1	11.0	81.1	11.4	0.368
	MMSE Total score	27.3	2.8	27.3	2.9	0.911
	MoCA Total score	23.6	3.9	23.6	4.3	0.984
	MDRS Total score	133.3	16.2	132.2	21.5	0.587
	Mild neurocognitive disorder	78	21.6%	49	18.8%	0.230
	Major neurocognitive disorder	47	13.0%	33	12.7	0.503
	*LARS total score*	*−20.4*	*10.8*	*−22.8*	*8.9*	*0.004*

NMSS	*NMSS cardiovascular*	*2.9*	*3.8*	*3.8*	*4.1*	*0.004*
	NMSS sleep problems	13.0	9.6	14.3	9.9	0.108
	*NMSS mood problems*	*12.4*	*14.3*	*15.3*	*12.3*	*0.016*
	NMSS hallucinations	1.5	4.1	1.5	3.3	0.976
	NMSS memory problems	6.2	7.1	6.4	7.1	0.686
	NMSS gastrointestinal problems	4.9	6.0	4.3	5.6	0.179
	NMSS urinary problems	10.0	9.1	11.2	10.2	0.130
	*NMSS sexual problems*	*2.9*	*5.8*	*1.8*	*5.2*	*0.022*
	*NMSS miscellaneous problems*	*3.8*	*4.8*	*5.5*	*6.3*	*0.000*
	*NMSS total score*	*57.4*	*41.2*	*64.1*	*41.1*	*0.045*

Health-related quality of life	EQ-5D VAS	64.8	20.1	62.4	20.1	0.135
	*EQ-5D index value*	*0.663*	*0.229*	*0.620*	*0.240*	*0.026*
	*PDQ-39 mobility*	*31.0*	*27.9*	*39.6*	*27.7*	*0.000*
	PDQ-39 ADL	29.3	25.0	26.8	24.8	0.221
	*PDQ-39 emotional well being*	*25.0*	*20.7*	*34.9*	*24.9*	*0.000*
	PDQ-39 stigma	23.2	24.5	25.1	27.3	0.370
	*PDQ-39 social support*	*11.0*	*15.4*	*13.8*	*16.8*	*0.038*
	PDQ-39 cognition	21.5	19.3	21.0	16.9	0.751
	*PDQ-39 communication*	*20.1*	*20.6*	*16.4*	*18.7*	*0.022*
	*PDQ-39 bodily discomfort*	*27.3*	*20.9*	*39.2*	*24.8*	*0.000*
	*PDQ-39 summary index*	*23.5*	*15.9*	*27.1*	*17.0*	*0.010*
	SES total score	75.0	16.2	74.1	19.4	0.636

For statistical analysis unpaired *t*-test was applied. In cases of items marked with ^*∗*^additionally Mann-Whitney test was also applied and yielded statistically significant differences.

Abbreviations: ACE = Addenbrooke Cognitive Examination; BDI = Beck Depression Inventory; EQ-5D = EuroQol Instrument 5 layer version; EQ-5D VAS = EuroQol Instrument Visual Analogue Scale; ESS = Epworth Sleepiness Scale; HAM-A = Hamilton Anxiety Scale; LARS = Lille Apathy Rating Scale; LED = levodopa-equivalent dosage; MADRS = Montgomery-Asberg Depression Rating Scale; MDRS = Mattis Dementia Rating Scale; MDS-UPDRS = The Movement Disorder Society-sponsored Unified Parkinson's Disease Rating Scale; MDS-UPDRS MC = Motor Complication part of MDS-UPDRS; MDS-UPDRS ME = Motor Examination part of MDS-UPDRS; M-EDL MDS-UPDRS = Motor-Experiences of Daily Living part of MDS-UPDRS; MMSE = Mini-Mental Status Examination; nM-EDL MDS-UPDRS = Non-Motor-Experiences of Daily Living part of MDS-UPDRS; MoCA = Montreal Cognitive Assessment; NMSS = Non-motor Symptoms Scale; PAS = Parkinson's Disease Anxiety Scale; PDQ-39 = Parkinson's Disease Questionnaire; PDSS-2 = Parkinson's Disease Sleep Scale 2nd version; UDysRS = Unified Dyskinesia Rating Scale.

**Table 3:** Impact of gender on impulse control disorders associated with Parkinson's disease.

		Sex				*p* value (chi-test)
		Male		Female		
		Count	Percentage	Count	Percentage	
Presence of any ICD	No ICD	31	8.6%	22	8.5%	0.850
	Yes ICD	78	21.6%	52	20.0%	

Punding	No	307	85.0%	223	85.8%	0.637
	Yes	54	15.0%	37	14.2%	

Compulsive eating	No	321	88.9%	228	87.7%	0.637
	Yes	40	11.1%	32	12.3%	

*Hypersexuality*	*No*	*343*	*95.0%*	*260*	*100.0%*	*0.000*
	*Yes*	*18*	*5.0%*	*0*	*0.0%*	

Pathological gambling	No	338	93.6%	251	96.5%	0.106
	Yes	23	6.4%	9	3.5%	

*Compulsive buying*	*No*	*338*	*93.6%*	*212*	*81.5%*	*0.000*
	*Yes*	*23*	*6.4%*	*48*	*18.5%*	

ICD: impulse control disorder.

## Abbreviations

ACE: Addenbrooke Cognitive Examination
BDI: Beck Depression Inventory
EQ-5D: EuroQol Instrument 5-layer version
EQ-5D VAS: EuroQol Instrument Visual Analogue Scale
ESS: Epworth Sleepiness Scale
HAM-A: Hamilton Anxiety Scale
ICD: Impulse control disorders
LARS: Lille Apathy Rating Scale
LED: Levodopa equivalent dosage
MADRS: Montgomery-Asberg Depression Rating Scale
MDRS: Mattis Dementia Rating Scale
MDS-UPDRS: The Movement Disorder Society-Sponsored Unified Parkinson's Disease Rating Scale
MDS-UPDRS MC: Motor Complication part of MDS-UPDRS
MDS-UPDRS ME: Motor Examination part of MDS-UPDRS
M-EDL MDS-UPDRS: Motor-Experiences of Daily Living part of MDS-UPDRS
MMSE: Mini-Mental Status Examination
nM-EDL MDS-UPDRS: Non-Motor-Experiences of Daily Living part of MDS-UPDRS
MoCA: Montreal Cognitive Assessment
NMSS: Non-motor Symptoms Scale
PAS: Parkinson's Disease Anxiety Scale
PDQ-39: Parkinson's Disease Questionnaire
PDSS-2: Parkinson's Disease Sleep Scale 2nd version
QUIP: Questionnaire for impulsive-compulsive disorders in Parkinson's disease
SES: Schwab-England Scale
UDysRS: Unified Dyskinesia Rating Scale.
